# Getting the most from after action reviews to improve global health security

**DOI:** 10.1186/s12992-019-0500-z

**Published:** 2019-10-10

**Authors:** Michael A. Stoto, Christopher Nelson, Rachael Piltch-Loeb, Landry Ndriko Mayigane, Frederik Copper, Stella Chungong

**Affiliations:** 1 Georgetown University Center for Global Health Science & Security, 3700 Reservoir Road, NW, Washington, DC 20057-1107 USA; 2 Harvard T.H. Chan School of Public Health, Boston, MA USA; 3 RAND Corporation and RAND Pardee Graduate School, Santa Monica, CA USA; 4 NYU College of Global Public Health and the Program on Population Impact Recovery and Resilience, New York, NY USA; 5 Core Capacity Assessment, Monitoring and Evaluation (CME) Unit Country Health Emergency Preparedness and IHR Department, WHO Health Emergencies Programme, World Health Organization, Geneva, Switzerland

**Keywords:** After action reviews (AARs), After action reports (AARs), Critical incident reviews, Public health emergencies, Public health preparedness, Systems improvement

## Abstract

**Background:**

After Action Reviews (AARs) provide a means to observe how well preparedness systems perform in real world conditions and can help to identify – and address – gaps in national and global public health emergency preparedness (PHEP) systems. WHO has recently published guidance for voluntary AARs. This analysis builds on this guidance by reviewing evidence on the effectiveness of AARs as tools for system improvement and by summarizing some key lessons about ensuring that AARs result in meaningful learning from experience.

**Results:**

Empirical evidence from a variety of fields suggests that AARs hold considerable promise as tools of system improvement for PHEP. Our review of the literature and practical experience demonstrates that AARs are most likely to result in meaningful learning if they focus on incidents that are selected for their learning value, involve an appropriately broad range of perspectives, are conducted with appropriate time for reflection, employ systems frameworks and rigorous tools such as facilitated lookbacks and root cause analysis, and strike a balance between attention to incident specifics vs. generalizable capacities and capabilities.

**Conclusions:**

Employing these practices requires a PHEP system that facilitates the preparation of insightful AARs, and more generally rewards learning. The barriers to AARs fall into two categories: concerns about the cultural sensitivity and context, liability, the political response, and national security; and constraints on staff time and the lack of experience and the requisite analytical skills. Ensuring that AARs fulfill their promise as tools of system improvement will require ongoing investment and a change in mindset. The first step should be to clarify that the goal of AARs is organizational learning, not placing blame or punishing poor performance. Based on experience in other fields, the buy-in of agency and political leadership is critical in this regard. National public health systems also need support in the form of toolkits, guides, and training, as well as research on AAR methods. An AAR registry could support organizational improvement through careful post-event analysis of systems’ own events, facilitate identification and sharing of best practices across jurisdictions, and enable cross-case analyses.

## Background

Globalization processes, including urbanization, changes in land use patterns, ecological change and biodiversity, vastly increased global commerce and travel, as well as increasing inequality and a lack of health system resilience have increased both the emergence of novel pathogens and their ability to cause cross-border threats to health [1]. In response, the World Health Organization (WHO) revised the International Health Regulations (IHR) in 2005 to ensure mutual accountability for health security [2]. This begins with the mandatory States Parties self-assessment annual reporting and the Voluntary External Evaluation processes using the Joint External Evaluation (JEE) tool that assess national preparedness capacities and to provide a more comprehensive picture of Member States in the implementation of the 13 IHR core capacities [3].

In this context, After Action Reviews (AARs) provide a means to observe how well preparedness systems perform in real world conditions and can help to identify – and address – gaps in national and global public health emergency preparedness (PHEP) systems [4]. AARs, along with simulation exercises (SimEx), assess the functionality of these capacities, both individually and working together in a coordinated and effective fashion. AARs also can help ensure that plans, processes, and other capacities are up to date and make the best possible use of limited resources. In an attempt to improve the overall state of AAR practice, the WHO has recently published the Country Implementation Guidance for voluntary After Action Reviews and Simulation Exercises under the WHO International Health Regulation Monitoring and Evaluation Framework (IHR MEF) [5].

The U.S. Army appears to have been the first to develop and institutionalize the AAR process (in the 1970s), and authored the first guidance for its implementation [6, 7]. Subsequently the approach was adopted by the Navy, Air Force, and Marines, and AARs are now required by regulation [8, 9]. Subsequently, the humanitarian response community, perhaps by virtue of working alongside the military in crisis response, adopted the practice of using AARs for organizational learning in disaster relief efforts. Organizations including World Vision have hosted conferences to establish internal and industry-wide lessons learned after major disasters to assess and improve performance and inform future responses, as they did after the Asian Tsunami in 2005 [10, 11].

AARs are now fairly common in PHEP. For over a decade they have been required of recipients of U.S. federal grants [12] and have frequently been conducted in Europe on major responses such as the 2017 Portugal fires [13], country- and EU-level responses to Ebola [14] and H1N1 [15, 16]. At the global level WHO has been promoting the use of AARs as a more science- or evidence-based approach to assessing effective IHR core capacities in “real-life” situations. Since the end of 2016, the WHO has supported more than 43 AARs globally, such as the Madagascar Plague AAR in July 2018 [17]. However, simply conducting AARs without meaningful *learning* from events can turn into a “box-checking” exercise.

Learning from actual events requires overcoming a number of challenges. First, the incidents that form the basis of AARs are singular, often rare, events that are usually unique in context and specifics. Thus, standard quality improvement (QI) techniques, which often rely on statistical analysis of repeated measures, are of limited use [4]. Second, the PHEP “system” is fragmented and its structure and function vary by location. As noted in the WHO “Whole of Society” approach, it includes public and private partners from the health and non-health sectors at the global, national, state and local levels, with each type of partner often playing different roles depending on the context and nature of the incident [18]. This complexity makes it difficult to know who should have done what, even after the event. Third, when done well, AARs can be time- and resource-intensive and often reveal uncomfortable truths.

Given these challenges, it is not surprising that the quality of AARs varies considerably. For instance, Savoia and colleagues (2012) analyzed AARs of responses to the 2009–2010 H1N1 pandemic and three hurricanes Ike (2008), Gustav (2008) and Katrina (2005) that appeared in the U.S. Department of Homeland Security’s Lessons Learned Information Sharing system (an online repository of AARs and best practices) [19]. Although there were many common themes, there was no consistency in how the capabilities were named or defined or what was included in each capability section. Similarly, participants at a workshop of U.S. federal, state, and local health officials who had prepared or reviewed AARs on the public health response to the 2009 H1N1 Pandemic found that these AARs varied widely in their intended uses, how they were prepared, and the extent to which they probed root causes [20]. Similarly, a recent analysis of 24 AARs identified both extensive variability in methods and a substantial divergence between real-world AAR practice and the standards described in AAR and qualitative research literature [21].

This review, intended for practitioners who conduct AARs, aims to build on and supplement the WHO guidance [22] by reviewing existing evidence on the effectiveness of AARs as tools for system improvement and by summarizing some key lessons about ensuring that AARs result in meaningful learning from experience. Our analysis and conclusions are drawn from the authors’ experience (spanning over 15 years) in conducting and reviewing AARs, researching effective AAR practices, and in developing tools for improving them. We cite an extensive literature on the subject, drawn from public health and other fields. But since much of this evidence does not appear in peer-reviewed journals, a structured systematic review would not have been effective. Perhaps because the AAR process began in the United States, most of the experience and evidence we cite is U.S-based.

Some of this literature uses the term Critical Incident Review rather than AAR, sometimes to indicate a more probing, thoughtful analysis than is seen in some AARs. In this commentary we use AAR to be consistent with the language of the IHR MEF, and describe best practices that can help ensure the critical analysis that we believe is necessary to make AARs effective. We also describe the need for a Critical Incident Registry, which would feature deeper analyses than in typical AARs. In the literature, AAR sometimes stands for “after-action report.” Because we want to emphasize the process, we focus on the review rather than the report in this analysis.

Our review begins with an appraisal of the evidence that AARs lead to system improvement. We then address best practices for conducting AARs, including choosing incidents that are ripe for learning, when to conduct AARs, who should be involved in the process, as well as how to conduct AARs, focusing on systems-thinking such as avoiding individual blame and probing for root causes. We end with a discussion of implementation issues, including overcoming barriers to conducting and reporting the results of AARs, the need to share results in a Critical Incident Registry, as well as to develop resources to aid in the conduct of effective AARs.

## Do AAR’s lead to system improvement?

AARs seek to create the conditions under which practitioners and stakeholders can use information collected to *improve* performance during future responses. We are not aware of any systematic research on the impact of AARs in public health emergency preparedness. However, a number of studies in other sectors and contexts provide evidence on the impact of the incident review process on individual and team performance, as well as organizational benefits [23]. Based on this limited evidence, there is reasonable justification to expect AARs could be an effective intervention in improving PHEP systems performance.

Tannenbaum and Cerasoli conducted a systematic review of findings from 46 studies [24]. Limiting their analysis to studies that reported on the impacts of AARs on “quantifiable aspects of performance” (e.g., in simulators, games, personnel records, self-ratings, performance appraisal ratings) they found that, on average, after action reports/debriefs improved effectiveness over a control group by approximately 25%. The results were similar across a wide variety of contexts, including teams versus individuals and medical versus non-medical situations. Another study that used survey data on 67 fire crews found that increases in the frequency of after-action reviews was associated with a stronger perceived safety climate [25]. A study of soldiers from two companies of the Israel Defense Forces taking a ground navigation course found added benefit from daily after-action reviews of both successes and failures, compared with those who reviewed only failures [26].

Both the United States Department of Veterans Affairs (VA) and the Joint Commission which monitors hospitals review incidents in their own form of after-action reporting. Each uses a systematic approach that incorporates root cause analysis into a review after a sentinel or adverse event has occurred where things did not go as expected. While limited evaluation has occurred of the effectiveness of the after-action reviews, at the VA, comparison of these reviews with prior approaches to reviewing adverse events showed a shift in the root causes identified, blaming individuals less and increasingly attributing the problem to systemic causes like communication and policies or procedures [27].

## Best practices for conducting after action reviews

Because of the recent emphasis on AARs and their success in other sectors, the remainder of this paper summarizes best practices and lessons learned about improving the quality of AARs as tools for learning and highlights some implications for practitioners and policy makers. The lessons address *what* kinds of incidents to review, *when* to do the reviews, *who* should be involved in the review, and especially, *how* the reviews should be conducted, including systematic and methodological approaches and considerations of generalizability. Lacking formal evidence, this section is based primarily on experience and professional consensus.

### Choosing incidents that are ripe for learning

Given the time and effort needed to conduct high-quality AARs, it is important to focus on incidents that are ripe for learning. Extremely large or severe incidents usually warrant an AAR if only because they affect large numbers of people and attract public attention. But smaller events that highlight important system characteristics, call into question key planning assumptions, or portend future trends can also provide important learning opportunities. AARs need not focus only on problems; good outcomes can be an opportunity for learning as well. Most incidents include a mix of good and bad outcomes anyway. Similarly, industries such as aviation have made great progress by reviewing “near misses” – small incidents, or even non-incidents, that could have been much worse under different circumstances [4].

Piltch-Loeb and colleagues have identified six considerations for selecting incidents for review [4].

1. public health played a significant – though not necessarily leading – role
2. the incident reflects a particular magnitude of morbidity or social disruption
3. the incident revealed particular vulnerabilities in response capabilities
4. it called into question systems behavior or beliefs
5. the incident helped to identify best practices, or
6. the incident captured the PHEP community’s attention or was otherwise meaningful for practitioners.

Similarly, but more specifically, the WHO guidance gives five criteria for initiating an AAR [5, 22]:

1. at least one of the 13 IHR core capacities is reviewed, validated or tested
2. the event was declared as a Public Health Event of International Concern (PHEIC), or was notified to WHO under the IHR (2005) Annex 2, or was a graded emergency under WHO Emergency Response Framework (level 2 or 3)
3. when the WHO Public Health Emergency Operations Centre was activated following the occurrence of a public health event, or due to an increased risk of occurrence
4. the event involved coordination and collaboration with sectors that do not routinely collaborate (e.g. chemical or radiological events, food safety event and natural disasters); or
5. when the AAR was recommended by WHO following an event that constitutes an opportunity for learning and performance improvement, which could include the above Piltch-Loeb considerations.

One of the challenges in analyzing actual events is that public health emergencies often play out over a long period – months rather than days or weeks. Many things happen during this period, making it difficult to know how to focus the analysis. One way to address this is to focus attention on “critical junctures,” phases in the incident that altered the response in a positive or negative way.

For example, in their analysis of the initial recognition of pandemic H1N1 influenza in Mexico and the U.S. in 2009, Zhang and colleagues created a timeline based on scientific literature, websites, news reports, key informant interviews [28]. This allowed the researchers to identify two critical junctures: (1) the identification of the novel pH1N1 virus in two California children and (2) Mexican health authorities’ recognition that a series of apparently unconnected respiratory disease outbreaks throughout Mexico were actually manifestations of pH1N1, which was later declared a Public Health Emergency of International Concern (PHEIC) by the WHO Director General following the recommendation of the IHR Emergency committee [29].

In-depth analysis of these events found that the identification of the California cases was made possible by expanded surveillance capacity, specifically an experimental surveillance system operated by the U.S. Navy. Similarly, the connection between the U.S. and Mexican outbreaks was made through a trilateral treaty that allowed the Mexican samples to be tested first in Canada and then in the U.S. as well as advanced in global communication systems as well as expectations under the IHR that potential PHEICs be reported. By focusing on these pivotal events, this analysis demonstrated the value of surveillance and notification capacities, as well as the capability to use them effectively, that are fundamental national state parties’ responsibilities under the IHR.

Another challenge lies in striking a balance between focusing on the details of specific incidents while at the same time probing for more generalizable lessons. The concepts of capacity and capability can provide a common terminology that allows researchers and practitioners to describe the details of specific incidents as examples of broader system functions that apply across times, places, and incident types. The U.S. for instance has identified a list of 15 public health preparedness capabilities [30] and 4 capabilities for hospitals and healthcare coalitions [31]. More recently, the European Centre for Disease Prevention and Control (ECDC), in turn, has adopted its own capacities and capabilities, derived from a logic model that includes *capacities* (the resources a PHEP system has to draw upon such as infrastructure, policies and plans, trained personnel) and response *capabilities* (the actions PHEP systems can take to detect, characterize, & respond to emergencies) (see Table 1).

**Table 1:** Response capabilities [32]

Detection and assessment	
• Surveillance & epidemiological monitoring	
• Incident recognition	
• Risk characterization	
• Laboratory analysis	
• Epidemiological investigation	
• Environmental monitoring	
Policy development, adaptation, and implementation	
• For infection control and treatment guidance	
• For population-based disease control	
• Communicating between national and subnational authorities and enforcing laws and regulations	
Health services	
• Preventive services	
• Medical surge	
• Management of medical countermeasures, supplies & equipment	
• Medical services for health care workers & emergency responders	
Coordination and communication	
• Crisis management	
• Communication with healthcare providers	
• Communication with emergency management, public safety, and other sectors	
• Communication with other public health agencies at the global, European, national, and subnational levels	
Emergency risk communication	
• Address communication inequalities	
• Generate dynamic listening and manage rumors	
• Communicate risk in an accurate, transparent and timely manner	
• Generate and maintain trust	

For example, an AAR of the response to the 2017 pulmonary plague outbreak in Madagascar using standardized WHO methodology [22] identified multiple challenges in terms of coordination and logistics; monitoring and laboratory; communication, social mobilization and community engagement; case management and infection prevention and control; and vector control, anti-reservoir and environment. Building on this analysis, the AAR identified 23 priority improvement activities, 9 of which aligned with recommendations from a JEE conducted in July 2017 [17]. A year later, the number of cases decreased by approximately 90% [33], suggesting that the implementation made an important difference in Madagascar. But what are the lessons for other countries? Many of the challenges and solutions related to the problem of identifying cases at the local level. This includes the availability, limitations, and improper use of tests; the dissemination and use of a standard case definition; the lack of standard operating procedures (SOPs) for reporting and notification; awareness among health workers; and training gaps. While the specifics will necessarily vary among countries, the need for an effective infrastructure to identify cases at the local level is likely to be generalizable.

### When to conduct AARs

Several researchers have noted that one of the most important mechanisms through which AARs can promote system improvement is by providing experiential learning opportunities in which individuals and groups engage actively around first-hand experiences and that serve to motivate them to do better in the future [24, 34, 35]. Thus, an important aspect of many AARs is an initial “hot-wash” or debrief of responders that come immediately or soon after the incident and that provides an opportunity to record facts and impressions and to capture initial learning as memories are fresh and before the pull of daily duties reduces organizations’ focus on the incident. In long lasting incidents, it can be useful to produce interim reports, perhaps at the end of each phase of the incident.

However, it is also important to assure that there is time for deeper reflection, which often comes with the passage of time. The hot-wash, therefore, is usually an integral part of an AAR, but is not by itself sufficient and should be followed by deeper analysis in the months that follow. Information collected during a hot-wash or debrief will be used during an AAR for deeper analysis for the collective sensemaking and organizational learning.

WHO recommends an AAR to be conducted within 3 months of the end of the event and/or of the response, when response stakeholders are still present and have clear memories of what happened [5]. Practically speaking, however, for some public health emergencies there is no hard end point, but rather an extended response and recovery period. Hurricane Maria struck Puerto Rico in September 2017, but the recovery was still under way more than a year later. In other circumstances, more time is needed to prepare the final AAR either because the event and consequently the analysis is complex, or time is needed for emotions to cool off enough so that a rationale analysis is possible. Having recently completed an AAR on a similar topic in another jurisdiction can facilitate rapid planning.

For instance, consider a disease outbreak that occurred in Alamosa County, Colorado, in 2008. In this incident, laboratory testing quickly confirmed *Salmonella typhimurium* as the responsible pathogen but it took almost two weeks to determine that contamination of the city’s public water supply was the source of exposure, which delayed efforts to stop transmission. An initial hot wash by public health officials focused on the response but did not address the causes of a 12-day delay in identifying the source of the contamination. Through a facilitated lookback meeting (see below) that brought together different responders after some time had passed, using a root cause analysis (RCA) framework, more causal pathways for delays – including interagency coordination and challenges in communication between health authorities and the agency responsible for the county’s drinking water – were identified. Over time and with deeper analysis, lessons learned shifted from fixing infrastructure to improving relationships and shared decision-making [36].

### Who should be involved in AARs

Consistent with WHO’s Whole of Society approach, the response to a critical incident involves a wide range of stakeholders. Effective analysis of incidents examines the critical event incident from multiple perspectives and objectives including the full range of stakeholders involved in the event. Bringing stakeholders to the table to participate in an incident review can be challenging due to concerns about blame, timing, or responsibility. However, one of the things that can make AARs effective as mechanisms of system improvement is that they (a) catalyze group dynamics that activate social control, social comparison, socialization, and bonding [34, 35, 37, 38], and (b) create venues in which groups can generate nuanced mental models that are shared by individuals playing different roles in the system. Indeed, an analysis of post-incident reviews in chemical plants finds that systems often fail because various operators and managers have different or conflicting mental models and assumptions, and that AARs provide a mechanism for identifying and resolving contradictions among them [39].

Two suggested methods to improve stakeholder engagement in the analysis of incidents include using a facilitated lookback or a peer assessment review. Facilitated lookback methodology developed by RAND to facilitate structured discussions with public health leaders and key staff [40]. A facilitated lookback uses a neutral facilitator and a no-fault approach. It brings together key stakeholders and responders in a meeting to probe dimensions of decisions and explore nuances in past decision-making. Rather than focusing on the response of an individual actor, the meeting focuses on decision-making and the shared experience around the event to solicit improvement strategies. A peer assessment review involves bringing in external peers in reviewing an incident. This option offers the potential for reliable and objective analyses by professionals familiar with PHEP and the particularities of the responding PHEP system. This also provides an effective way to share best practices across jurisdictions.

### How to conduct AARs: the importance of systems-thinking

There are also important lessons about the manner in which AARs are conducted, once an incident has been selected and participants selected. There is widespread agreement that the purpose of AARs is to improve systems, not blame individuals or organizations when things go wrong. Placing blame in this way can make those who were involved with the response unwilling to participate freely, thus reducing the quality of the analysis. More importantly, this approach misses an opportunity to learn about problems with the response system that, if not addressed, could cause problems in future events. Focusing on systems rather than individual actions, also help “open up” participants who might otherwise be concerned about personal reprisals [6, 24, 34]. As such, policymakers should be careful in attaching incentives to AARs. One approach is to hold recipients of grants accountable for doing AARs, but not for the results of the analysis, which is the approach taken by the US Department of Health and Human Services in promoting SimExs and AARs for healthcare coalitions [30]. Empirical support for this comes from a study that assessed learning by aviation pilots from near-misses, both in narrative reports filed by experienced pilots after actual dangerous aviation incidents and in laboratory experiments in which college students operated a flight simulator under different conditions of organizational accountability [41]. The authors reviewed narratives provided by the pilots and found that counterfactual thinking, which they regard as a key element in AARs, was less prevalent when the pilots believed they would be held accountable for the near-miss.

AARs should be structured in a way that moves beyond identification of symptoms of problems to system-level *root causes.* The Ebola virus cases that emerged in Dallas and New York City in the fall of 2014 illustrate this point. In Dallas, a Liberian resident visiting relatives in Dallas came to a hospital emergency department with Ebola symptoms, but it was not until four days later that the local and state health departments mounted a full public health response. In New York, a physician who had been treating Ebola patients in West Africa developed a fever and within hours, an aggressive public health response began the same day. While there are many reasons for the slower response in Dallas, a careful analysis of the case (Table 2) suggests that one contributing factor is that the Dallas hospital did not act like it was part of a public health *system* (e.g., by sharing information and engaging key partners in a timely manner), with responsibilities to the community as well as its patients. In New York, on the other hand, the Department of Health and Mental Hygiene has a long history of collaborating with the city’s hospitals, and in this case they prepared as a system, including conducting “mystery patient” drills [43].

**Table 2:** Ebola virus in Dallas and New York City

Although global public health systems had been slow to respond to the first cases in West Africa earlier in the year, by September Ebola stories were prominent in the U.S. media and professional publications. In addition, the Centers for Disease Control and Prevention (CDC), state, and local health departments throughout the country alerted hospitals, which in turn distributed this information to first line providers. On Thursday, September 25, a Liberian resident (Mr. D) visiting relatives in Dallas, Texas developed symptoms consistent with Ebola and sought care at the Texas Health Presbyterian Hospital emergency department (ED). Despite telling one of the nurses that he was from Liberia, he was sent home. On Sunday, September 28, Mr. D returned to the same hospital by ambulance, with more severe symptoms. This time he was consider a potential Ebola case and was “isolated in the ED.” Samples were not sent for testing to CDC and the Texas Department of State Health Services until Monday, and positive results were received on Tuesday, September 30, at which point a public health response was initiated. During this 4-day period, two nurses were infected with Ebola. Mr. D died on September X, and the nurses survived. On Wednesday, October 15, Dr. S, a physician who had been treating Ebola patients in Guinea with Médecins Sans Frontières (MSF) returned home to New York City and in the following days travelled throughout the city using public transportation. On Thursday, October 23, following MSF protocols, took his own temperature and reported a low-grade fever. A few hours later he was taken by a special ambulance to an isolation ward that had been prepared Bellevue Hospital Center. Two of Dr. S’s friends were quarantined, and by that evening the Mayor, the New York City health commissioner, and others held a press conference outlining the public health response. Dr. S was treated and survived, and there were no additional cases. It is clearly inappropriate to directly compare the two cases – an uninsured traveler from Liberia and a physician trained by MSF – and the first case is always more difficult. One can, however, examine each system’s response. Although problems with the EHR may have contributed to the failure to diagnose Mr. D’s case the first time he came to the hospital in Dallas [42], there were additional delays in taking precautions to prevent transmission to others in the hospital and in sending samples to be tested, due in part to a lack of protocols. The Texas Health Presbyterian Hospital did not act like it was part of a public health *system*, with responsibilities to the community as well as its patients. In New York, on the other hand, not only did MSF have protocols in place, but the Department of Health and Mental Hygiene worked with city’s hospitals to prepare as a system, including conducting “mystery patient” drills [43].	

One common way to identify root causes is to ask “why” up to five times (with the number of times depending on need and circumstance) to drive down to the core of a problem and identify fixes that are likely to be lasting [44]. For example, AARs must ask not only how quickly cases were detected or how many vaccines were delivered but also how and why systems performed as they did, and what changes could improve system performance in the future. This team has previously proposed several steps that can improve RCA within a broader incident analysis. The steps used to conduct a RCA as well as an example of how these steps apply to an incident are shown in Table 3. The example refers to the Salmonella outbreak in Alamosa, CO referenced above [36].

**Table 3:** Root Cause Analysis steps and example

1. Define the story arc by summarizing the context and pivotal nodes (events, decisions, time points) when events could have unfolded differently and could have led to a substantially different outcome. 2. Identify the public health system’s major organizational goals or objectives in responding to the incident, including which PHEP Capabilities and IHR (2005) core capacities that were stressed. 3. Identify the major response challenges that had a qualitative impact on permitting achievement of the public health system’s goals or at least had the potential to do so. 4. Define the immediate causes of the challenges and the factors that contributed to the challenges, whether modifiable (within the jurisdiction’s influence) or not modifiable (not within the jurisdiction’s influence); note pre-event decisions and factors beyond the system’s control. 5. Identify factors that, if not addressed, are likely to limit the public health system in future incidents. With these steps in mind, RCA can help those conducting the AAR to include the deepest level of analysis within their review.	

Analyzing critical incidents in systems terms often requires rethinking notions of methodological rigor. Given the singular nature of PHEP events and the complexity of systems responses, reliance on statistical analysis of large populations of cases is not only difficult to do, but may narrow the analysis in a way that misses important system properties. For instance, beyond knowing the numbers on non-pharmaceutical distribution, morbidity, mortality or cost, effective learning requires deeper exploration of why the incident unfolded the way it did to produce such outcomes. Drawing on the social science literature, especially Gilson [45], Table 4 summarizes methods for improving the rigor of qualitative research that can strengthen the AAR practice. The 11 validity-enhancing recommendations for AARs proposed by Davies and colleagues address many of the same points [21]. Stoto and colleagues [46] illustrate specific considerations to improve analysis such as timing, perspective, and drawing on root cause analysis. Stoto [47] describes how these methods were used to conduct a rigorous, multi-faceted analysis of the public health system response to 2009 H1N1.

**Table 4:** Ensuring Rigor in Case Study and Qualitative Data Collection and Analysis [45, 46]

• Prolonged engagement with the subject of inquiry. Health policy and systems research tends to draw on lengthy and perhaps repeated interviews with respondents and/or days and weeks of engagement at a case study site.	
• Use of theory. Theory is essential to guide sample selection, data collection, analysis, and interpretive analysis.	
• Case selection. Purposive selection allows earlier theory and initial assumptions to be tested and permits an examination of “average” or unusual experience.	
• Sampling. It is essential to consider possible factors that might influence the behavior of the people in the sample and ensure that the initial sample draws extensively across people, places, and time. Researchers need to gather views from a wide range of perspectives and respondents and not allow one viewpoint to dominate.	
• Multiple methods. For each case study site, best practice calls for carrying out two sets of formal interviews with all sampled staff, patients, facility supervisors, and area managers and conducting observations and informal discussions.	
• Triangulation. Patterns of convergence and divergence may emerge by comparing results with theory in terms of sources of evidence (e.g., across interviewees and between interview and other data), various researchers’ strategies, and methodological approaches.	
• Negative case analysis. It is advisable to search for evidence that contradicts explanations and theory and then refine the analysis accordingly.	
• Peer debriefing and support. Other researchers should be involved in a review of findings and reports.	
• Respondent validation. Respondents should review all findings and reports.	
• Clear report of methods of data collection and analysis (audit trail). A full record of activities provides others with a complete account of how methods evolved.	

## Implementation

The WHO’s recent inclusion of AARs in its International Health Regulation Monitoring and Evaluation Framework (IHR MEF) is an important step in increasing the prevalence of AARs. Beyond this, additional steps may be necessary to ensure that AARs are of high quality and that lessons from individual communities and jurisdictions are shared broadly with others. Employing the best practices described in this analysis requires a PHEP system that facilitates the preparation of insightful AARs, and more generally rewards learning.

In many countries, the barriers to after action reviews fall into two categories. First, there are sometimes concerns about the cultural sensitivity and context, liability, the political response, and national security. In addition, after-action reviews are constrained by staff time and the lack of experience and the requisite analytical skills. Ensuring that AARs fulfill their promise as tools of system improvement will require ongoing investment and a change in mindset. The first step should be to clarify that the goal of AARs is organizational learning, not placing blame or punishing poor performance. Based on experience in other fields, the buy-in of agency and political leadership is critical in this regard. As Stufflebeam has said of evaluation, the “purpose is not to prove, but to improve” [48].

Even well-prepared AARs are often not widely shared with those who could benefit from them. For instance, responders to the Boston Marathon bombing learned valuable lessons from previous events in Israel and elsewhere [49]. Unfortunately, such sharing is often the exception rather the rule, and depends on personal connections among responders. Sectors such as aviation have benefited greatly from the creation of registries that collect incident reports [50]. For instance, in order to enhance transparency, trust and mutual accountability among Member States and partners, the WHO promotes the sharing of AAR as well as SimEx results using a minimum reporting template in the country implementation guidance [5]. The standardized reporting template includes explicit linkages to existing IHR MEF instruments that emphasizes voluntary evaluation of functional capacities as demonstrated by real or simulated events. WHO plans to make the information collected through the reporting template publicly available, a step towards developing a lessons-learned database for public health emergencies.

Moving beyond this, a Critical Incident Registry for PHEP could provide a database of incident reports filed by public health agencies that responded to a critical incident can drive organizational improvement through careful post-event analysis of systems’ “own” events, facilitate identification and sharing of “best practices” across jurisdictions, and enable cross-case analyses to identify contexts and mechanisms that determine success [4]. CIR entries could be based on countries’ internal AARs, but focused on issues likely to be of interest elsewhere. For instance, as illustrated in the plague example above, the CIR entry would focus on the need for an effective infrastructure to identify cases at the local level rather than the specific problems experience by Madagascar and the country-specific solutions they adopted.

Entries in the registry should have a common structure that facilitates analysis of individual incidents and cross-case analysis; a searchable, structured summary that includes a list of the PHEP capabilities tested; a timeline of pivotal events in the incident; and an analysis of PHEP system’s role in enough detail to understand why particular mechanisms worked in that context. Entries could be coded by such factors as incident type, capabilities involved, levels of organizations involved (i.e., local, regional, national, international), which could promote analysis. Reports in the registry would have to meet minimum quality standards, based on the points discussed here and the ECDC’s 11-point validity tool [21]. An additional benefit of such a registry is that it could allow analysis to identify common patterns across incidents and learn from structured comparisons among cases.

A Critical Incident Registry can also be useful to involve individuals with expertise in PHEP systems, but who were not part of the response, in the preparation of AARs. As well as simply providing help, this also can improve credibility of the findings and trust in process. These could be peers from other jurisdictions or faculty, students, or staff from schools of public health or other academic units. The involvement of peers from other countries has also contributed to the success of the JEE process and a series of country-level preparedness analyses based on the response to Ebola conducted by ECDC [51, 52], and this bodes well for the involvement of peers in AARs.

Finally, national public health systems need support in the form of toolkits, guides, and training, as well as research on AAR methods. For instance, WHO, ECDC, and others could develop and disseminate tools, templates, training materials, and checklists that lead users through the process of conducting high-quality AARs [53] and build upon ongoing efforts by WHO and ECDC to develop an AAR registry [5, 21, 54]. In addition, practitioners, policymakers and journal publishers could work together to give awards to recognize and incentivize particularly high-quality AARs – and those that include honest and thorough-going analysis of response gaps and system weaknesses – and publish them in scientific/professional journals or other outlets.

## Conclusions

Effective after-action reviews are designed to provide practitioners and policymakers with knowledge and tools they can use to learn from experience and improve public health plans and responses. Empirical evidence from a variety of fields suggests that the practice can improve performance on simulated and real-world tasks. While direct evidence on public health responses is not yet available, this analysis shows that AARs hold considerable promise as tools of system improvement for PHEP. Our review of the literature and over 15 years of practical experience demonstrates that AARs are most likely to result in meaningful learning if they focus on incidents that are selected for their learning value, involve an appropriately broad range of perspectives, are conducted with appropriate time for reflection, employ systems frameworks and rigorous tools such as facilitated lookbacks and root cause analysis, and strike a balance between attention to incident specifics vs. generalizable capacities and capabilities. Using these approaches can help ensure that countries efforts to fulfill their obligations under the IHR (2005) contribute not only to enhancing their own preparedness but also to generating lessons relevant to others. And since the use of AARs is still relatively new in PHEP, we anticipate that additional experience with this process with lead to advances in AAR methods as well.

## Data Availability

Not applicable.

## Abbreviations

AAR: After action review
ECDC: European Centre for Disease Prevention and Control
IHR ME: International Health Regulation Monitoring and Evaluation Framework
IHR: International Health Regulations (2005)
JEE: Joint External Evaluation
PHEIC: Public Health Event of International Concern
PHEP: Public health emergency preparedness
RCA: Root cause analysis
SimEx: Simulation exercise
SPAR: States Parties self-assessment annual reporting
VA: United States Department of Veterans Affairs
WHO: World Health Organization

## References

[1] Lederberg J, Hamburg MA, Smolinski MS. Microbial threats to health: emergence, detection, and response: National Academies Press; 2003. 25057653

[2] World Health Organization. International health regulations (2005): World Health Organization; 2008.

[3] World Health OrganizationH. Monitoring and evaluation framework joint external evaluation tool (JEE tool)- second. Edition Geneva, Switzerland; 2018.

[4] Piltch-Loeb R, Kraemer JD, Nelson C, Stoto MA (2014). A public health emergency preparedness critical incident registry. Biosecur Bioterror.

[5] World Health Organization. Country implementation guidance: after action reviews and simulation exercises under the international health regulations 2005 monitoring and evaluation framework (IHR MEF). World Health Organization; 2018.

[6] Garvin DA. Learning in action: a guide to putting the learning organization to work: Harvard Business Review Press; 2003.

[7] Salter MS, Klein GE (2007). After action reviews: current observations and recommendations.

[8] United States Army Office of Regulations. Army Lessons Learned Program (ALLP). 2006.

[9] United States Marine Corps. MCO 3504.1 Marine Corps Lessons Learned Program (MCCLP). 2006.

[10] Rawal V, Fautin C, Moore J-L, Kalonge S, Walden VM, Bhattacharjee A (2005). Multi-agency evaluation of tsunami response: India and Sri Lanka. CARE international, world vision (WV), Oxfam GB, Catholic relief services (CRS).

[11] Sexton RM, Isobel A. Comparative study of after action review (AAR) in the context of the southern Africa. Crisis. 2003.

[12] United States Department of Homeland Security (2007). Homeland security exercise and evaluation program: the department.

[13] Neto M, Pimentel J, Morais A, Santos C, Ferreira AJ, Ferreira PL. Preparação das famílias para fazer face a emergências e catástrofes: avaliação após o incêndio de 2017 ocorrido nos concelhos de Pedrógão Grande. Figueiró dos Vinhos e Castanheira de Pera; 2018.

[14] European Union Conference. Lessons learned for public health from the Ebola outbreak in West Africa – how to improve preparedness and response in the EU for future outbreaks. Luxembourg; October 2015. p. 12–4.

[15] Fineberg H, Aavitsland P, Aditama T, Bino S, Carmo E. Implementation of the international health regulations (2005): Report of the review committee on the functioning of the international health regulations (2005) and on pandemic influenza a (H1N1) 2009. Geneva: World Health Organization. Geneva: World Health Organization. p. 2011.

[16] Hine D (2010). The 2009 influenza pandemic: an independent review of the UK response to the2009 influenza pandemic.

[17] World Health Organization. Revue Après Action de la Réponse D’urgence á la Flambée Épidémique de Peste Pulmonaire. Organisation mondiale de la Santé; 2018 2 AU 6 Juillet 2018.

[18] World Health Organization (2017). WHO guidance for surveillance during an influenza pandemic.

[19] Savoia E, Agboola F, Biddinger PD (2012). Use of after action reports (AARs) to promote organizational and systems learning in emergency preparedness. Int J Environ Res Public Health.

[20] Stoto MA, Nelson C, Higdon MA, Kraemer J, Singleton C-M (2013). Learning about after action reporting from the 2009 H1N1 pandemic: a workshop summary. J Public Health Manag Pract.

[21] Davies R, Vaughan E, Fraser G, Cook R, Ciotti M, Suk JE. Enhancing reporting of after action reviews of public health emergencies to strengthen preparedness: a literature review and methodology appraisal. Disaster Med Public Health Prep. 2018:1–8. 10.1017/dmp.2018.82 30220258

[22] World Health Organization. Guidance for after action review (AAR): World Health Organization; 2019.

[23] Scott C, Dunn AM, Williams EB, Allen JA (2015). Implementing after-action review systems in organizations: key principles and practical considerations.

[24] Tannenbaum SI, Cerasoli CP (2013). Do team and individual debriefs enhance performance? A meta-analysis. Hum Factors.

[25] Allen JA, Baran BE, Scott CW (2010). After-action reviews: a venue for the promotion of safety climate. Accid Anal Prev.

[26] Ellis S, Davidi I (2005). After-event reviews: drawing lessons from successful and failed experience. J Appl Psychol.

[27] Wu AW, Lipshutz AK, Pronovost PJ (2008). Effectiveness and efficiency of root cause analysis in medicine. Jama..

[28] Zhang Y, Lopez-Gatell H, Alpuche-Aranda CM, Stoto MA (2013). Did advances in global surveillance and notification systems make a difference in the 2009 H1N1 pandemic?–a retrospective analysis. PLoS One.

[29] World Health Organization. Swine Influenza Statement [press release] Geneva, Switzerland. 2009.

[30] United States Centers for Disease Control and Prevention. Public health preparedness capabilities: national standards for state and local planning, March 2011: Centers for Disease Control and Prevention, Office of Public Health Preparedness and Response 2011.

[31] United States Centers for Disease Control and Prevention. 2017–2022 Health care preparedness and response capabilities. 2016.

[32] Stoto MA, Nelson C, Savoia E, Ljungqvist I, Ciotti M (2017). A public health preparedness logic model: assessing preparedness for cross-border threats in the European region. Health security..

[33] World Health Organization. Actions taken to reduce plague burden in Madagascar and recent Cases Show Decline [press release] Geneva, Switzerland, 5 June 2019 2019.

[34] Morrison JE, Meliza LL. Foundations of the after action review process: Institute For Defense Analyses Alexandria Va; 1999.

[35] Lipshitz R, Popper M, Friedman VJ (2002). A multifacet model of organizational learning. J Appl Behav Sci.

[36] Piltch-Loeb Rachael, Kraemer John, Nelson Christopher, Savoia Elena, Osborn David R., Stoto Michael A. (2018). Root-Cause Analysis for Enhancing Public Health Emergency Preparedness. Journal of Public Health Management and Practice.

[37] Brown JS, Duguid P (1991). Organizational learning and communities-of-practice: toward a unified view of working, learning, and innovation. Organ Sci.

[38] Ron N, Lipshitz R, Popper M (2006). How organizations learn: post-flight reviews in an F-16 fighter squadron. Organ Stud.

[39] Carroll JS (1998). Organizational learning activities in high-hazard industries: the logics underlying self-analysis. J Manag Stud.

[40] Aledort JE, Lurie N, Ricci K, Dausey DJ, Stern S (2006). Facilitated look backs: a new quality improvement tool for management of routine annual and pandemic influenza.

[41] Morris MW, Moore PC (2000). The lessons we (don't) learn: counterfactual thinking and organizational accountability after a close call. Adm Sci Q.

[42] Upadhyay DK, Sittig DF, Singh H (2014). Ebola US patient zero: lessons on misdiagnosis and effective use of electronic health records. Diagnosis..

[43] Foote M, Daver R, Quinn C (2017). Using “mystery patient” drills to assess hospital ebola preparedness in new York City, 2014-2015. Health security.

[44] Croteau RJ. Root cause analysis in health care: tools and techniques. Joint Commission Resources. 2010.

[45] Gilson L, Hanson K, Sheikh K, Agyepong IA, Ssengooba F, Bennett S (2011). Building the field of health policy and systems research: social science matters. PLoS Med.

[46] Stoto MA, Nelson CD, Klaiman T. Getting from what to why: using qualitative methods in public health systems research. Academy Health Issue Brief. 2013.

[47] Stoto MA, Higdon MA. The public health response to 2009 H1N1: a systems perspective: Oxford University Press; 2015.

[48] Stufflebeam DL, Shinkfield AJ (1985). Stufflebeam’s improvement-oriented evaluation. Systematic evaluation: springer.

[49] Biddinger PD, Baggish A, Harrington L, d'Hemecourt P, Hooley J, Jones J (2013). Be prepared—the Boston Marathon and mass-casualty events. N Engl J Med.

[50] Billings C, Lauber J, Funkhouser H, Lyman E, Huff E (1976). NASA aviation safety reporting system.

[51] Kandel N, Sreedharan R, Chungong S, Sliter K, Nikkari S, Ijaz K (2017). Joint external evaluation process: bringing multiple sectors together for global health security. Lancet Glob Health.

[52] European Centers for Disease Control and Prevention. Ebola emergency preparedness in EU member states – conclusions from peer-review visits to Belgium, Portugal and Romania. Stockholm; 2015.

[53] World Health Organization. After action review Geneva, Switzerland: World Health Organization; 2019. [Available from: https://extranet.who.int/sph/after-action-review].

[54] Learning from Critical Incidents Toolkit [Available from: https://www.hsph.harvard.edu/preparedness/toolkits/critical-incidents/].

